# Efficacy improvement in searching MEDLINE database using a novel PubMed visual analytic system: EEEvis

**DOI:** 10.1371/journal.pone.0281422

**Published:** 2023-02-09

**Authors:** Jong-Chan Lee, Brian J. Lee, Changhee Park, Hyunjoo Song, Chan-Young Ock, Hyojae Sung, Sungjin Woo, Yuna Youn, Kwangrok Jung, Jae Hyup Jung, Jinwoo Ahn, Bomi Kim, Jaihwan Kim, Jinwook Seo, Jin-Hyeok Hwang

**Affiliations:** 1 Department of Internal Medicine, Seoul National University Bundang Hospital, Seongnam, Korea; 2 College of Medicine, Seoul National University, Seoul, Korea; 3 Department of Computer Science & Engineering, Seoul National University, Seoul, Korea; 4 Department of Internal Medicine, Seoul National University Hospital, Seoul, Korea; 5 School of Computer Science & Engineering, Soongsil University, Seoul, Korea; 6 Bang & Ock Consulting, Inc., Seoul, Korea; Ege University, Faculty of Medicine, TURKEY

## Abstract

PubMed is the most extensively used database and search engine in the biomedical and healthcare fields. However, users could experience several difficulties in acquiring their target papers facing massive numbers of search results, especially in their unfamiliar fields. Therefore, we developed a novel user interface for PubMed and conducted three steps of study: step A, a preliminary user survey with 76 medical experts regarding the current usability for the biomedical literature search task at PubMed; step B is implementing EEEvis, a novel interactive visual analytic system for the search task; step C, a randomized user study comparing PubMed and EEEvis. First, we conducted a Google survey of 76 medical experts regarding the unmet needs of PubMed and the user requirements for a novel search interface. According to the data of preliminary Google survey, we implemented a novel interactive visual analytic system for biomedical literature search. This EEEvis provides enhanced literature data analysis functions including (1) an overview of the bibliographic features including publication date, citation count, and impact factors, (2) an overview of the co-authorship network, and (3) interactive sorting, filtering, and highlighting. In the randomized user study of 24 medical experts, the search speed of EEEvis was not inferior to PubMed in the time to reach the first article (median difference 3 sec, 95% CI -2.1 to 8.5, *P =* 0.535) nor in the search completion time (median difference 8 sec, 95% CI -4.7 to 19.1, *P =* 0.771). However, 22 participants (91.7%) responded that they are willing to use EEEvis as their first choice for a biomedical literature search task, and 21 participants (87.5%) answered the bibliographic sorting and filtering functionalities of EEEvis as a major advantage. EEEvis could be a supplementary interface for PubMed that can enhance the user experience in the search for biomedical literature.

## Introduction

Literature searching is a crucial step in conducting scientific research, preparing presentation material, and selecting study topics. PubMed (https://pubmed.ncbi.nlm.nih.gov/), which has been developed and is administered by the National Center for Biotechnology Information (NCBI) at the United States National Library of Medicine (NLM) [1], is free database in the biomedical field accessed by millions of users from around the world [1]. PubMed covers most of the published biomedical literature and more than three million of its cases receive clicks by 2.5 million users per day [1].

The advantages of PubMed can generally be described as follows: (1) an enormous amount of biomedical records including Index Medicus and Non-Index Medicus [2]; (2) access to related databases such as BioProject (formerly known as the Genome Project), other genetics, proteomics, and the Medical Subject Headings (MeSH) database [3–5]; (3) an easy and intuitive user interface (UI) [5]; (4) free accessibility to the advanced search mode [5]; (5) individual optimization using a My NCBI account [5]; (6) multiple filters displayed on the left side of the browser [6]; (7) a continuously evolving search algorithm [1]; and (8) various additional functions that provide user friendliness [7]. These characteristics of broadness and specificities have resulted in the fact that almost all meta-analysis research includes PubMed as a search strategy.

The PubMed database provides a simple search interface. However, the search interface supports only a limited set of data analysis methods, and it is difficult to interpret and explore through a massive literature dataset. The recent flooding of article publication and journals has made it even more difficult for medical researchers to conduct accurate searches for key articles or to learn about a subject effectively. Users can narrow the search space to the most relevant matches by successive text-based queries with a multi-bibliographic criteria syntax. Nevertheless, this task requires a highly skilled query construction ability and the domain knowledge of the search field [5]. Therefore, conducting a massive literature search task only with a text-based query language can be time-consuming and inefficient.

**Fig 1:**
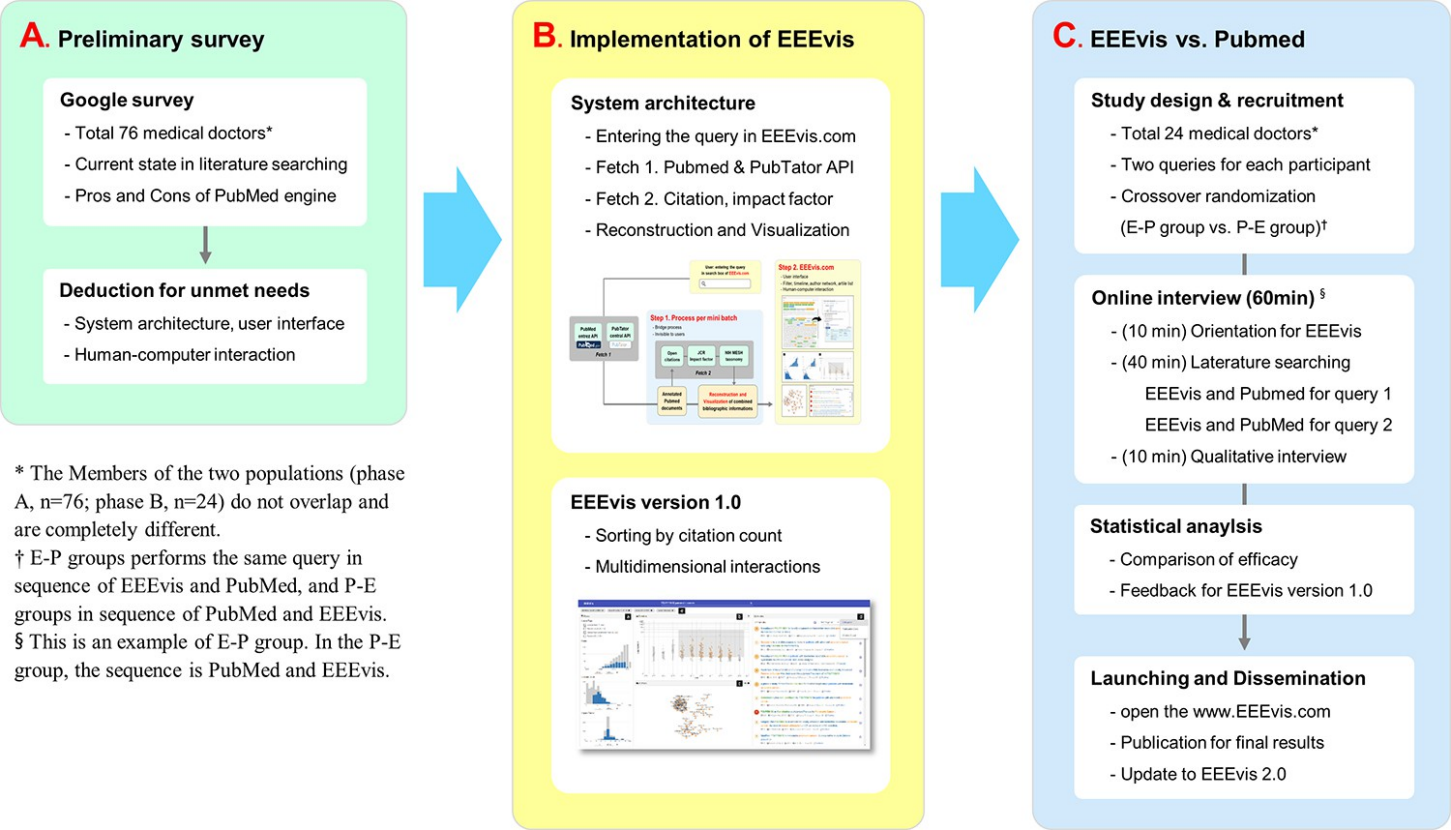
Study scheme. * The members of the two populations (phase A: n = 76; phase B: n = 24) did not overlap and were completely different. † The E-P group performed the same query in the sequence of EEEvis and PubMed, and the P-E group performed the query in the sequence of PubMed and EEEvis. § This is an example of the E-P group.

In this study, we developed a novel interactive visual analytic system for biomedical literature search called EEEVis (Easy and Efficient Evidence visual analytics). The study processes were performed in three steps; (A) a preliminary user survey regarding the current usability and the literature search task requirements of PubMed, (B) an implementation of the opinions from the user survey to the novel literature search system development, and (C) a randomized user study comparing the usability of PubMed and the novel search system to see if the implementations had been well accomplished and how the comparable the performances between PubMed and the novel system (Fig 1).

## Materials and methods

### A. Preliminary survey

We conducted a Google survey of 76 MDs between January and May 2019. The survey questions were categorized into three sections, as follows: (1) baseline information for the research career, (2) questions for current use patterns of PubMed or other literature search interfaces, and (3) additional requirements for a literature search task. The Google survey questions are summarized in S1 Table.

### B. Implementation of EEEvis

**Fig 2:**
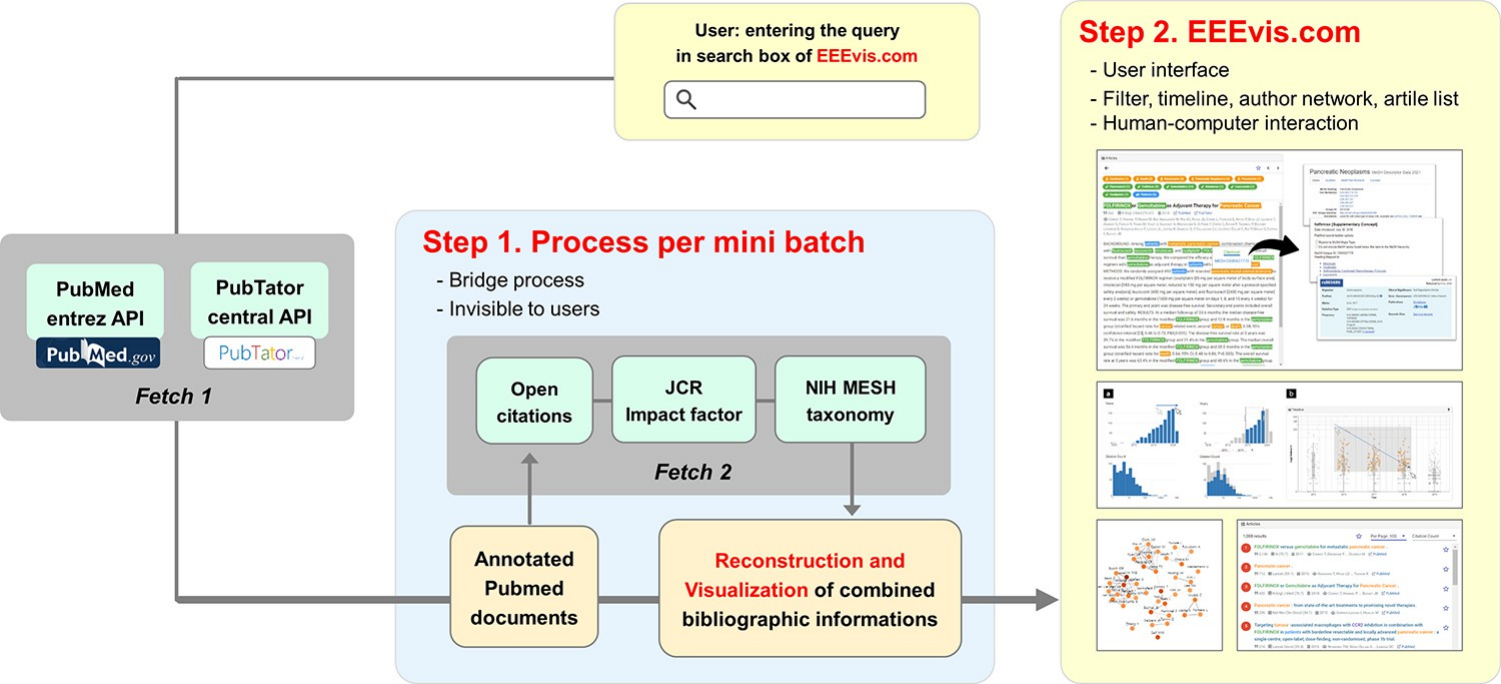
System architecture for EEEvis.

EEEvis is a web-based interactive visual analytic system for biomedical literature search that employs a client–server architecture (Fig 2). The client is a web application into which users input PubMed syntax-based search queries (https://pubmed.ncbi.nlm.nih.gov/help/) and the results are fetched from the server. The web application is implemented using Angular (https://angular.io/), whereas the charts are implemented using Plotly.js (https://plotly.com/javascript/) and Vega (https://vega.github.io/‌vega/).

The server is a RESTful web service implemented in Python that fetches the PubMed search results of the query using PubMed Entrez Programming Utilities API [8]. After retrieving the search results, the server annotates the results with PubTator Central biomedical concept annotations [9], citation counts from the OpenCitations (https://opencitations.net/) and NIH MeSH databases (https://www.nlm.nih.gov/mesh/meshhome.html), and impact factors from Journal Citation Reports (https://jcr.clarivate.com/jcr/home).

However, larger search results will lead to a longer query time and slower user response. To avoid this limitation and to support responsive user interaction, the fetch and annotation process is divided into a series of mini-batches, and the responses are returned to the client progressively.

Unlike other user interfaces, the major enhancement of EEEvis is that it supplies not only the basic bibliographic information of each paper but also the citation count of each article and the impact factor of the journal in an integrated way, and it is implemented with human-computer interaction technique.

### C. Randomized user study

We conducted a randomized user study of 24 MDs between November to December 2020. This population was completely different from that of the preliminary user survey. All participants were first randomly assigned to one of two groups and a crossover randomization method (1:1) using a random number table was adopted for random allocation.

The two groups were as follows: (1) the E-P group, which performed the same query in the sequence of EEEvis and PubMed, and (2) the P-E group, which performed the same query in the sequence of PubMed and EEEvis.

**Fig 3:**
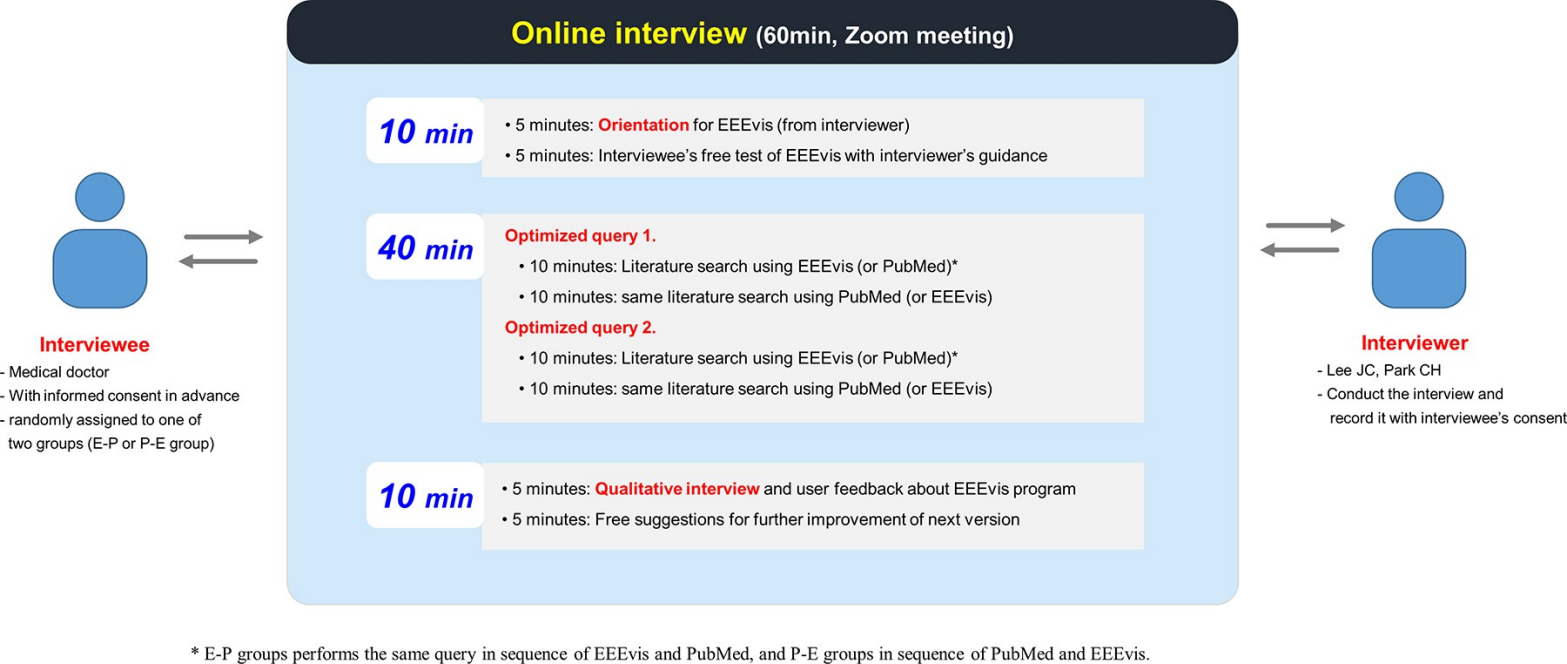
Schematic flow for randomized user study. * The E-P group performed the same query in the sequence of EEEvis and PubMed, and the P-E group performed the query in the sequence of PubMed and EEEvis.

Once the participants had signed the consent form, the actual study was conducted as a 1:1 online Zoom meeting (Zoom Video Communications, Inc., California, USA) between the interviewer and interviewee. The interview was conducted for a total of 60 minutes, and it consisted of 10 minutes of orientation and free use, 40 minutes of actual literature searching, and 10 minutes of user feedback (Fig 3).

In the first 10 minutes, as the participants had not been exposed to EEEvis before, the interviewer taught them how to use EEEvis for 5 minutes, following which they could freely use the system and test it for the remaining 5 minutes. If any question arose during use, the interviewer immediately provided an answer.

For the 40 minutes of literature searching, two optimized queries were provided according to the subspecialty of the interviewee. The first query was presented on the assumption that when participants prepare for a presentation at a conference or seminar, they find main reference articles on the subject. The interviewee could select up to 5 suitable papers for their requirements and performed a paper search using EEEvis or PubMed for a maximum time of 10 minutes. For the following10 minutes, the same query was executed using another search program that was not previously used. If the same process was repeated twice, the previous 10 minutes could be considered as learning about the corresponding query, which could lead to a bias (i.e., practice effect) whereby the results appeared to be better in the later 10 minutes. We randomly assigned the entire group to either the E-P or P-E group to correct this bias.

The second query was also optimized according to the subspecialty of the interviewee. In this case, when an academic thesis is written and a specific sentence is used, the researcher is searching for a reference article that can serve as a citation for that sentence. EEEvis and PubMed were assigned the same time of 10 minutes each and the order of the search interfaces was equally assigned according to the E-P or P-E group. The two queries that were presented for each field were provided blindly by assistant or associate professors in the corresponding subspecialty.

In the final 10 minutes, user feedback was provided based on open questions, and the results were qualitatively analyzed and summarized.

Once all 24 participants had been interviewed, two researchers (Sung HJ and Woo SJ) analyzed the video according to the standardized format, whereas two other researchers (Lee JC and Park CH) cross-checked the video. The overall analysis results were divided into two parts. The first part was related to the phase of entering search keywords, during which the time to achieve the optimal search strategy and loading time were measured as specific variables. The second part was the stage after the search results were presented, whereby the size of the search results, the number of selected articles, time to reach the first article, and time to complete the article selection were measured and analyzed. The existing assistant and associate professors who submitted the query evaluated how effectively the finally determined papers met the requirements and fit the topic. The papers were graded into three categories: (1) appropriate, (2) suboptimal, and (3) inappropriate; subsequently, the results were aggregated and analyzed. Finally, the results of the user feedback were classified as advantages in comparison to PubMed and suggestions for further improvement in the subsequent version according to their frequency.

This study was approved by institutional review board of Seoul National University Bundang Hospital (IRB No. B-2011/648-303). The risk of this study was determined as minimal risk. The participants were recruited after approval of the study. The informed consents of the participants for the preliminary survey were collected along with the survey. The informed consents of the participants for the user study were collected as written documents before the initiation of any process.

#### C.1. Statistical analysis for Randomized user study

To compare the efficacy of EEEvis and PubMed, we analyzed all the videos of 24 participants, and defined the variables as follows: (1) Time to reach optimal search strategy (sec) indicates the time from when a user inputs a search query in the search box until the user finally determines the desired query using Boolean symbols and keywords; (2) Loading time refers to the time from when the final query is entered and the Enter key is pressed until the final result list appears on the screen. (Check: EEEvis refers to the time until the final result list appears up to 1000 on one screen, and PubMed refers to the time until up to 20 appear on one screen); (3) Time to reach optimal search strategy (sec) is the total number of papers finally displayed for each query, which indicates whether the query was appropriate (excluding too broad or too narrow); (4) Number of selected articles per each query refers to the number papers of which the participant finally concluded were the target papers; (5) Time to reach the first article (sec) is the time from the moment the result screen starts to appear until the moment that the first target paper is found; (6) Time to complete article selection (sec) refers to the time taken from the moment of finding the first paper to the moment of finding the last paper, and it is an indicator of how closely the results that participants are looking for are gathered. (7) Number of optimal or suboptimal selections of articles means when the senior specialist in each field evaluates the list of target articles of the participant and judges that the result is very appropriate (optimal) or acceptably enough (suboptimal). For example, if a particular participant finds 5 papers and 3 of them are appropriate, 1 is acceptable enough, and 1 is inappropriate, the proportion of ‘optimal selection’ is 60% and the proportion of ‘optimal plus suboptimal selection’ is 80%.

This step C is different from a traditional randomized controlled trial. We used the term randomization because randomization between participants (E-P vs P-E) was done. However, the term ‘controlled’ was excluded because each query was modified to suit the specialized field and it cannot be said that all variables were controlled. Also, since the word ‘trial’ can be confused with a traditional clinical trial for patients, we used the expression ‘user study’.

All the ranges of continuous variables were shown in interquartile range (IQR). Since all variables did not satisfy the assumption of normality, we used Wilcoxon sign-rank test as the statistical analysis in this randomized user study. Because this design is different from the traditional cross-over design, we did not modify the Wilcoxon sign-rank test.

## Results

### A. Preliminary survey

#### A.1. User survey

We conducted an online questionnaire with medical doctors (MDs) who held professional qualifications in various clinical departments and had experience in medical research to derive the requirements of the biomedical literature search task. A total of 76 participants completed the questionnaire and the baseline demographic findings of the participants are summarized in Table 1.

**Table 1:** Demographic findings of preliminary survey.

Variables	Number (%) (n = 76) *
Age	
30–34	28 (36.8)
35–39	34 (44.7)
40–44	11 (14.5
45 or more	3 (3.9)
Specialty	
Gastroenterology	50 (65.8)
Neuropsychiatry	6 (7.9)
Internal medicine	3 (3.9)
Cardiology	2 (2.6)
Emergency medicine	2 (2.6)
Family medicine	2 (2.6)
General surgery	2 (2.6)
Pulmonology	1 (1.3)
Orthopedic surgery	1 (1.3)
Radiology	1 (1.3)
Pathology	1 (1.3)
Rheumatology	1 (1.3)
Plastic surgery	1 (1.3)
Neurosurgery	1 (1.3)
Thoracic surgery	1 (1.3)
Laboratory medicine	1 (1.3)
Year of obtainment of medical license	
2000 or before	2 (2.6)
2001–2005	8 (10.5)
2006–2010	43 (56.6)
2011–2015	23 (30.3)
Duration of exposure to PubMed (year)	
0–4	5 (6.6)
5–9	36 (47.4)
10–15	28 (36.8)
16 or more	7 (9.2)
Number of involved research articles as an author	
0	2 (2.6)
1~2	23 (30.3)
3~5	27 (35.5)
6~10	10 (13.2)
11~15	9 (11.8)
16 or more	5 (6.6)

* The population of the preliminary survey (n = 76) and that of the randomized user study (n = 24) are totally different.

Participants were recruited using document announcements, and medical specialists with experience in using PubMed and writing papers were allowed to apply. Among the 76 participants, 34 (45%) were between the ages of 35 and 39, and 28 (37%) were aged 30 to 34. Gastroenterologists constituted 50 (66%) of the participants. A major portion (93%) of the participants had been using PubMed for at least 5 years, and over 97% of the participants had published at least one medical article as a lead author.

**Fig 4:**
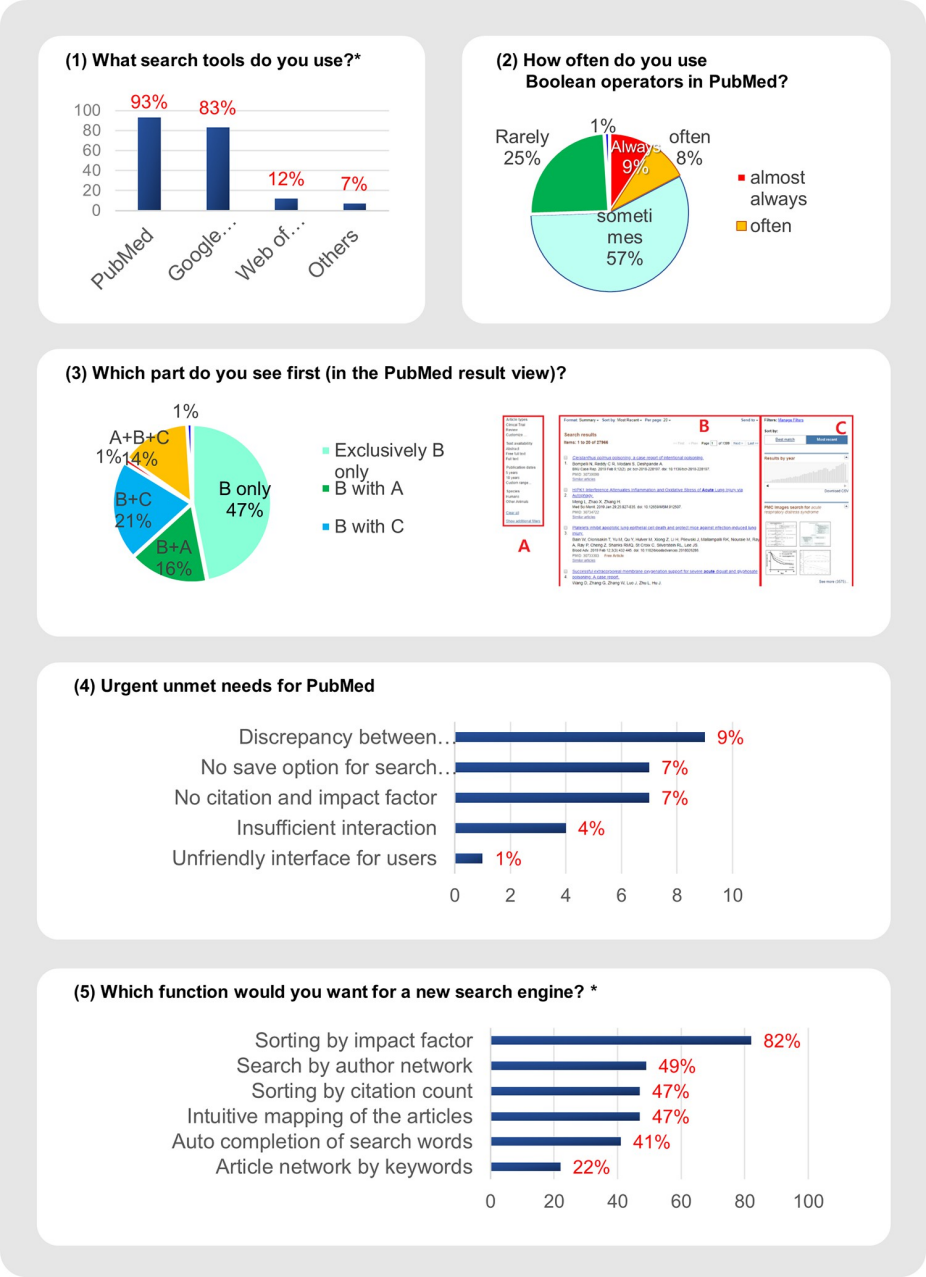
Results of preliminary survey. * Multiple choice was possible up to three answers.

As illustrated in Fig 4, the questionnaire consisted of the following five questions: (1) Which search tools do you use? (multiple possible responses); (2) How often do you use Boolean operators in PubMed?; (3) Which part do you see first (in the PubMed results view)?; (4) What are your urgent needs for PubMed?; and (5) Which function would you like in a new search engine? (multiple possible responses). Because the literature on the user experience of PubMed could not be found even with the methodology of systematic review, we developed the questions of preliminary Google survey through repeated meetings of authors at the study design phase.

In question (a), the most commonly used medical literature search tools were PubMed (93%), followed by Google or Google Scholar (83%). In question (b), Boolean operators such as AND, OR, and NOT were used by 74% of participants. In the primary screening process for the result window of PubMed, in question (c), 47% of participants selected the “body” section only. Users who screened all parts of the body and both sidebars constituted 14% of all participants.

In question (d), 28% of the participants reported the demands and requests to enhance the results of PubMed. The most submitted reply was to reduce the discrepancy between the search purpose and results. Finally, in open-ended question (e), the answers were submitted in the order: sorting by impact factor (82%), searching by author network (49%), sorting by citation count (47%), intuitive mapping of articles (47%), auto-completion of search words (41%), and article network by keywords (22%).

#### A.2. Design requirements

The preliminary survey aided in providing an understanding of the design requirements for a biomedical literature search system [10–14]. Based on the results, we determined the following requirements that should be supported by a biomedical literature search system:

- R1: Construct advanced search queries through direct manipulation [15].
- R2: Present an overview for the bibliographic information.
- R3: Present the trend and importance of the articles.
- R4: Present the bibliographic relationship with the co-author network.
- R5: Provide filtering and sorting functions to narrow the search.

### B. Implementation of EEEvis

#### B.1. System overview

**Fig 5:**
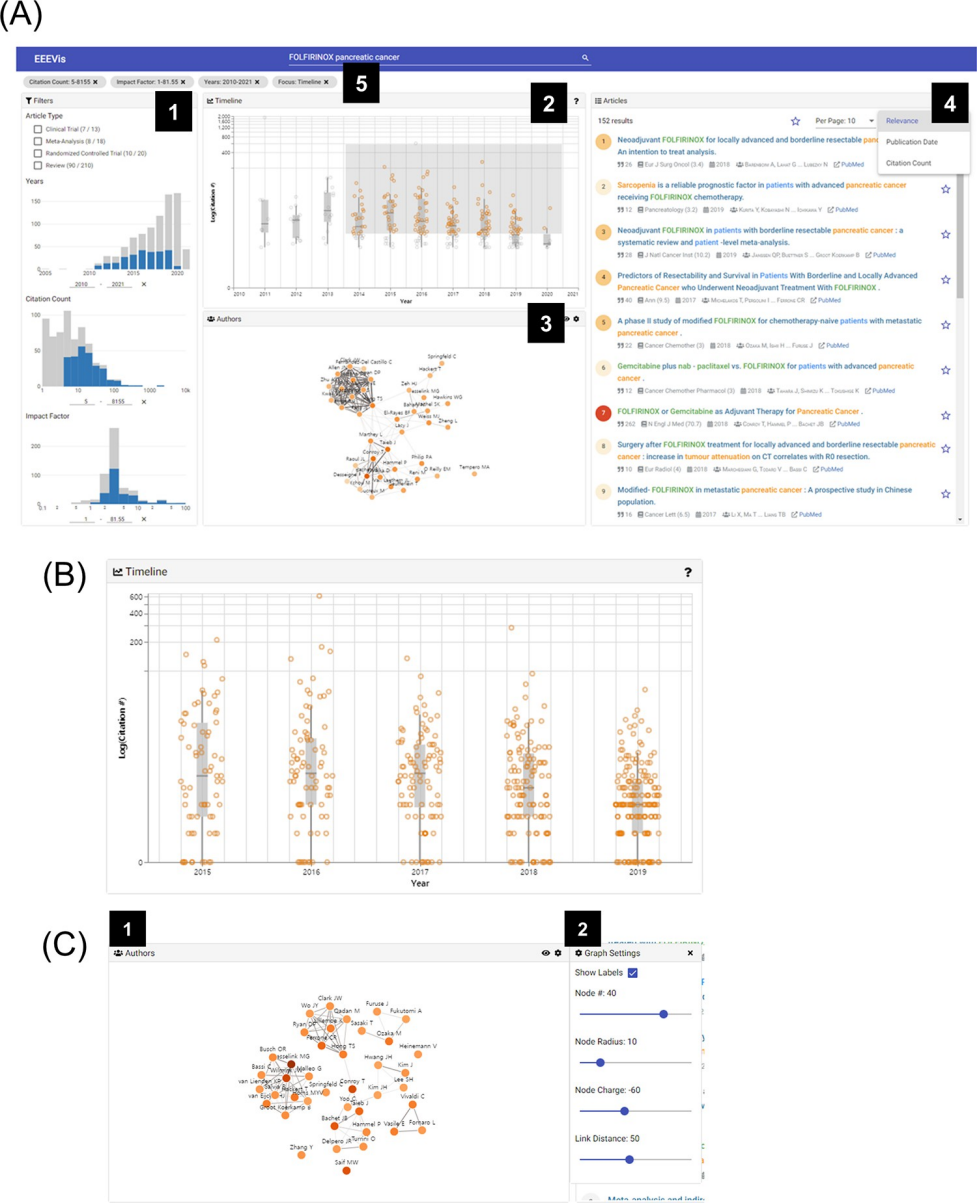
EEEvis interfaces. (A) Overview of EEEvis interfaces: (1) Bibliography Filters, (2) Timeline View, (3) Co-authorship Network View, (4) Article List & Detail View, (5) Filter Status Bar. (B) Timeline View. A series of boxplots on an ordinal timeline x-axis. The boxplot is a standard Tukey boxplot (the median, first quartile, and third quartile). The y-axis represents the citation counts with a logarithmic scale. (C) Co-authorship Network View. (1) This view represents the co-authorship network among the most published authors of the targeted subset with a force-directed node-link graph. The numbers of authorships and co-authorships are visually encoded into the node color and link strength. (2) Users can adjust the graph properties with the controls in the optional configuration side panel.

We designed and implemented a web-based visual analytic search tool known as EEEvis to assess and enhance the biomedical literature search results from the PubMed database. Through EEEvis, users can create a search query through direct manipulation that supports the PubMed search query syntax and visualize the results. However, while the PubMed web search interface presents the results in a page-based list and fetches only a limited number (10 ~ 200) of articles on each page, EEEvis fetches the abstract data of the complete search result set progressively (up to 10,000 results) and hands out an overview of the result set. EEEvis splits the fetch process into a series of tasks of fetching partial mini batches to overcome the fetch time delay and progressively updates the interface on each batch. This approach is similar to the progressive visual analytics (PVA) method that allows users to explore partial data analysis results in integrated and interactive visualizations during the execution [14]. Users can interactively explore the PubMed search results that are annotated with citation data and biomedical concept tags in an interface consisting of four coordinated views: (1) Bibliography Filters, (2) a Timeline View, (3) a Co-authorship Network View, and (4) an Article List & Detail View (Fig 5A).

#### B.2. Bibliography filters

This view provides an overview of the bibliographic features: the article type, year of publication, citation count, and impact factor. Each feature is mapped to a visual representation based on its data type. The visualizations are coordinated using the brushing and linking [16,17] technique so that users can filter a user-interest subset of the search query result with specific values or ranges of the feature in a visualization and all other linked visualizations also immediately reflect the filtering result (S1A Fig).

The article types, which are categorical values, are listed with the number of corresponding articles. The list supports multiple selections using a checkbox interface. A bar chart is employed to represent discrete values, such as the publication year, whereas histograms are used to represent continuous values, such as the citation count and impact factor. The continuous values are binned into 20 bins, which are calculated to have similar physical widths on the x-scale. As the citation count and impact factor usually exhibit skewness towards small values in a vast range, the x-axes are implemented on a logarithmic scale. The bar charts and histograms support interactive x-axis range selection (brushing) by dragging an area on the chart or inserting specific values into the text boxes below the x-axis. The gray bars represent the number of articles in the search query result and the blue bars represent the intersection of all filters (linking), thereby constituting the user-interest subset. Every filter is displayed with chip buttons in the filter status bar (Fig 5A). The other three coordinated views display the user-interest subset.

#### B.3. Timeline view

Users can already assess the annual trend of the number of articles per year using the year of publication histogram in the bibliography filters. However, as the citation count is also an important metric in literature searches, several tasks require the identification of the temporal trends of citation counts. Thus, we plot a series of boxplots according to the year of publication to reveal the annual trends (Fig 5B).

The x-axis is an ordinal timeline of each year of publication and the y-axis is the logarithmic scale of the citation counts. Every box plot represents the statistical data of the articles that are published each year. The box plots are in the form of a standard Tukey boxplot that displays the median, first quartile, and third quartile. The whisker ranges from the smallest to largest data within the range [Q1–1.5 * IQR, Q3 + 1.5 * IQR], where Q1 and Q3 are the first and third quartiles, and IQR is the interquartile range (Q3—Q1). Every article is encoded as a circular data point and jittered over the box plot to display the actual citation counts and distribution.

As the citation count is also correlated with the age of the article, the use of a single global range filter may lead to the omission of recent important articles or noise from many uninteresting old research papers. Thus, we implement a secondary filter function known as “Focus” in this view to dynamically explore a particular subspace of the time by citation count space. Users can focus on any timespan or citation count range by dragging a rectangular area on the chart (S1B Fig). The data points that correspond to the focus subset are color-coded in orange, but the complementary ones are in gray. These secondary filters are also displayed in the filter status bar with the prefix “Focus.” As a secondary brushing and linking technique, the Author Network View and Article List View also reflect this focused subset.

#### B.4. Co-authorship network view

The analysis of bibliometric networks [18–21], such as co-authorship or co-citation networks, has always been of major interest in the literature analysis domain. We implement an interactive force-directed node-link graph to reveal the co-authorship network, and to identify the hubs and authorities of the user-filtered subset (Fig 5C).

The node-link graph is constructed using the top-k publishing authors. The default value of the k-value, which is the number of nodes, is 40. The number of articles by each author is color-coded into the color of each node using a sequential orange color scheme. The links between the author nodes indicate co-authorship between the authors, whereas the width and saturation of each link represent the strength of the link. In this view, users can identify the leading authorities of the user-interest subset, highly connected subgroups, hub nodes of the subgroups, and bridge authors among the subgroups.

However, the graph properties, namely the number of nodes, node radius, node charge strength, and link distance, may need to be adjusted to reveal the network topology efficiently. Users can configure these properties in real time using the Graph Settings panel to monitor the network topology. Users can select each node and link to focus on the articles by an author and the articles to which authors contributed together. As the Co-authorship Network View, Timeline View, and Article List View are coordinated views, they all reflect this focused subset.

#### B.5. Article list & detail view

The articles of the search result are presented in Fig 5A. The list responds to the filter and focus interactions, and displays the user-interested subset of the articles. Users can sort the list according to several options: PubMed relevance [22], publication date, and citation count. Each list item includes the title, citation count, publication journal, impact factor, publication year, and authors. In an item, a circular glyph is placed ahead of the title, which is color-coded using an orange color scheme. The glyph color encodes the citation count of the article, which serves as an indicator of popular articles. Users can bookmark specific articles of interest by toggling the star-shaped button that follows each item. The list can be switched between displaying all articles or bookmarked articles by toggling the star-shaped button in the list toolbar.

The title is annotated with biomedical concepts using the PubTator Central application programming interface (API) [9]. PubTator Central parses natural language and recognizes biomedical named entities [23]. A total of six biomedical concept categories are annotated using different colors: genes, diseases, chemicals, mutations, species, and cell lines.

**Fig 6:**
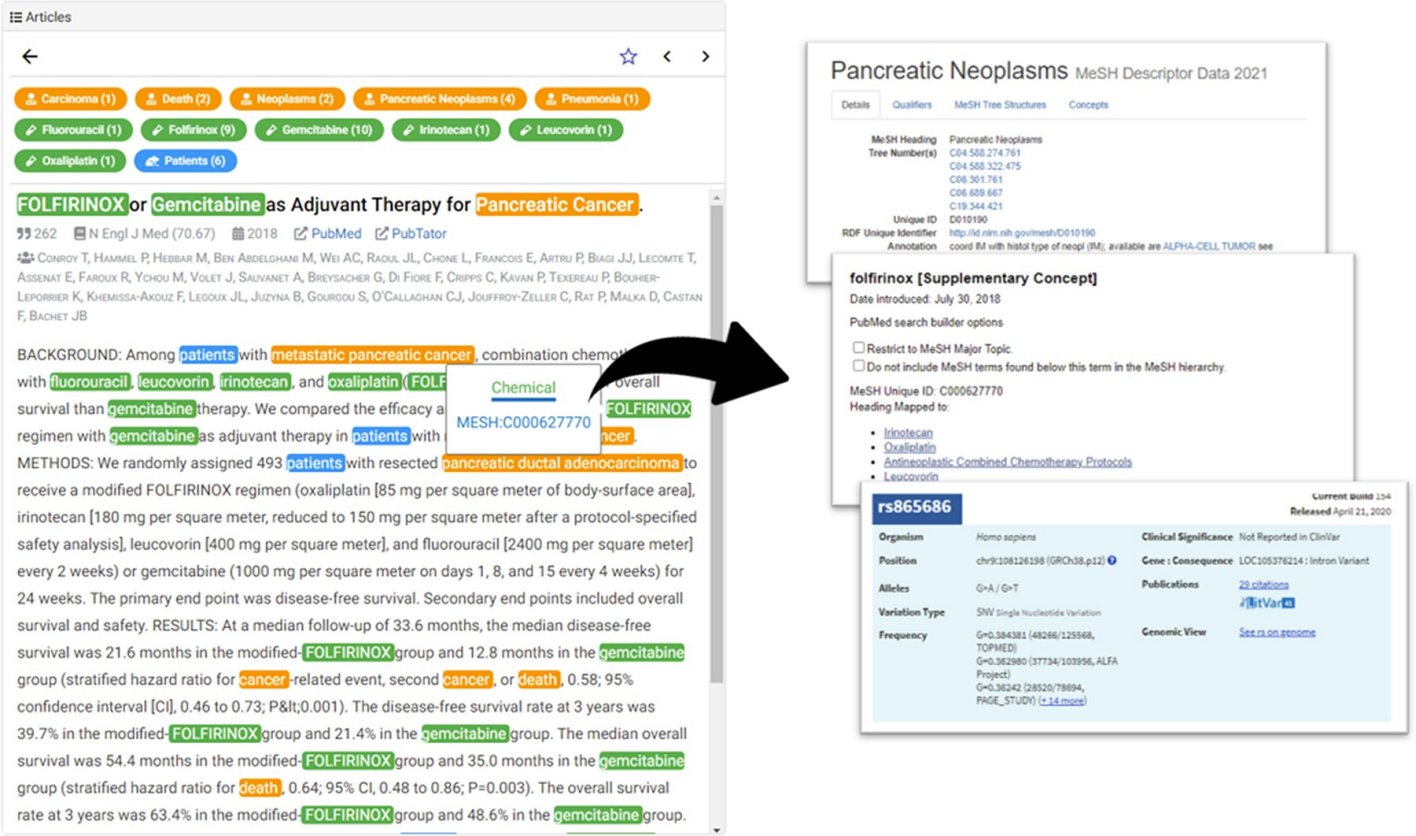
Article list & detail view. The Detail View displays the title, authors, citation counts, publishing source and data, external links to PubMed and PubTator, the abstract, and the PubTator annotations. Users can click on each annotation, which provides external links to the information of the biomedical entity.

When a user selects an article from the list or clicks on an article point from the Timeline View, the List View is transformed into the Detail View (Fig 6), which displays the annotated title, abstract, and more detailed information of the biomedical concept tags. A list of the concepts that appear in the title and abstract text is placed above the title so that users can comprehend the main keywords without reading the entire text. Users can also click on a tag and identify the ID of each biomedical term, such as MeSH term or NCBI Taxonomy IDs. The detailed information of each biomedical concept is provided, with a link to an external database.

### C. Results of user study

#### C.1. Demographic findings

We conducted a randomized user study using 24 MDs with various subspecialties to evaluate the effectiveness of the system in the medical field. As indicated in Table 2, all of the participants were in the first or second year of their fellowship and were specialists who were actively conducting medical research. The mean age was 36 years, and 33% of the participants were female. The participants had obtained a doctor’s license and had written an average of three papers as a first author. The participants had a median of four years of experience using PubMed; however, they had no exposure to EEEvis prior to the randomized user study. The population of this randomized controlled study (n = 24) was completely different from that of the preliminary survey (n = 76).

**Table 2:** Demographics findings of randomized user study.

Variables	Values* (n = 24) †
Age (year)	36 (33–38)
Gender	
Female	8 (33.3)
Male	16 (66.7)
Research career	
Years after medical college graduate	7 (5–9)
Published articles as a main author	3 (1–6)
Exposure to search browser	
Previous exposure to EEEvis	0 (0.0)
Previous exposure to PubMed	100 (100.0)
Duration of exposure to PubMed (years)	4 (2–6)
Subspecialty of fellowship	
Gastroenterology (non-HBP)	3 (12.5)
Gastroenterology (pancreatobiliary)	3 (12.5)
Gastroenterology (hepatology)	4 (16.7)
Oncology	2 (8.3)
Hematology	2 (8.3)
Cardiology	1 (4.2)
Pulmonology	1 (4.2)
Nephrology	1 (4.2)
Surgery, HBP	1 (4.2)
Surgery, gastric	1 (4.2)
Surgery, orthostatic	1 (4.2)
Surgery, thoracic	1 (4.2)
Pediatrics	1 (4.2)
Obstetrics	1 (4.2)
Anesthesiology	1 (4.2)

* Data are demonstrated as median (range) or number (%).

† The population of the randomized controlled user test (n = 24) and that of the preliminary survey (n = 76) are totally different.

EEEvis, medical and healthcare evidence visualizer; HBP, hepato-biliary-pancreas.

#### C.2. Comparison of efficacy

Assuming that E is EEEvis and P is Pubmed, all queries were searched identically in the order of E-P or P-E. Each participant was assigned two queries which has same sequence in the same participants. That is, some participants searched in the order of EPEP and other participants in the order of PEPE, and each participant’s assignment between EPEP and PEPE was determined by randomization. Following the randomization in the sequence of PubMed and EEEvis, two queries were provided to each participant (S2 Table). The search efficiency of which is compared in Table 3. None of the participants had experienced EEEvis prior to this study, which means that this was the first exposure to EEEvis for every participant. However, the participants had an average of 4 years of experience in using PubMed. The median numbers of articles that were listed as query results were 1,423 (EEEvis) and 1,446 (PubMed). The median times to reach the first targeted article were 93 (36 to 125) and 90 (35 to 119) seconds, respectively. The median times to complete the search were 298 (140 to 415) and 306 (102 to 397) seconds, respectively (*P* = 0.771). Whether the search results of this thesis were properly evaluated was determined by the scoring of external experts in each field, and the correct answer rate for search relevance was 87.9% vs. 90.5% (*P* = 0.637), respectively.

**Table 3:** Comparison of efficiency in literature searching.

	EEEvis	PubMed	P-value
Phase of entering search keywords			
Time to reach optimal search strategy (sec)	82 (10–119)	114 (11–184)	*0*.*039*
Loading time (sec)*	12 (8–18)	2 (1–2)	–
Phase after showing the results			
Number of listed articles per each query	1,423 (684–2,205)	1,446 (712–2,428)	*0*.*822*
Number of selected articles per each query	4 (2–5)	4 (2–5)	*0*.*537*
Time to reach the first article (sec)	93 (36–125)	90 (35–119)	*0*.*535*
Time to complete article selection (sec)	298 (140–415)	306 (102–397)	*0*.*771*
Quality of searched literature			
Sum of selected articles of overall 48 queries ^†^	N = 207	N = 199	–
Number of optimal selections of articles ^§^	182 (87.9)	180 (90.5)	*0*.*637*
Number of optimal plus suboptimal selections ^§^	197 (95.1)	192 (96.5)	*0*.*715*

Data are demonstrated as median (interquartile range) or number (%).

* The loading time of EEEvis is the duration of complete demonstration of all the results (1000 or more literature), whereas that of PubMed is the duration of dynamic demonstration of the first page results (initial 20 literature). Therefore, P-value was not presented in this row.

† Two queries were given per person out of a total of 24 participants.

§ Individual faculties in each subspecialty refereed the appropriateness of the article selection.

#### C.3. User feedback for EEEvis

During the last 10 minutes of online interview, the participants were asked open questions regarding the advantages and disadvantages of EEEvis and for suggestions for further improvement in the future version (Table 4). A total of 21 (87.5%) participants mentioned the sorting and filtering by the citation count and impact factor as an advantage. Increasing the accuracy and granularity of the filtering interaction (58.3%) and reducing the search loading time (45.8%) were the most frequent suggestions for further improvement in the future version. Finally, 22 of 24 participants (91.7%) responded that they are willing to use EEEvis as their first choice for a biomedical literature search task.

**Table 4:** Feedback for EEEvis version 1.0.

Advantages compared to PubMed	No (%)
Sorting and filtering by citation count and impact factor	21 (87.5)
Clean and intuitive interface	13 (54.2)
Article bookmark function	9 (37.5)
Author interaction plot	4 (16.6)
Accurate search output of important and target articles	3 (12.5)
MeSH term keyword tagging	1 (4.2)
Quick access to abstract	1 (4.2)
Suggestion for further improvement in next version	No (%)
Mouse dragging sensitivity in filter section	14 (58.3)
Loading time of whole demonstration of time	11 (45.8)
Save and export article bookmarks	8 (33.3)
Not suitable interface for mobile screen	3 (12.5)
Messy MeSH term keyword tagging	3 (12.5)
No advanced search function, thereby, impossible to apply to meta-analysis	2 (8.3)
Misspellings even a little results in wrong results	2 (8.3)
No exclude option	1 (4.2)
Difficult to modulate author interaction plot	1 (4.2)
Abstracts are not visible in the first place	1 (4.2)
No related or cited by articles	1 (4.2)
No direct full text link	1 (4.2)
Unable to save search options	1 (4.2)
Willingness to use EEEvis as the first choice for literature search	No (%)
Yes	22 (91.7)
No	2 (8.3)

## Discussion

We have presented the literature searching tool called EEEvis, a novel interactive visual analytic system for the biomedical literature search task. We compared the search performance of EEEvis and PubMed with medical researchers and evaluated their investigated the user feedbacks. The search performance measured as the time to obtain the appropriate search results was comparable between the two systems, despite EEEvis being new to the subjects. Also, in the qualitative questionnaire after user study, the feedback of participants showed considerably favorable trends toward EEEvis. For the reproducibility of statistical analysis, three links to access all the raw data are available at the S3 and S4 Tables.

We reflected the design requirements from the preliminary survey into EEEvis. The responses to the post-user-study questionnaire show the users found the best strength of EEEvis is to be the sorting and filtering functions (R5) of the impact factor and citation counts (R2). The runner-up response was the intuitive interface (R1), so we believe users found the system easy to use. Despite there being no significant difference between the task completion time of PubMed and EEEvis, users answered a preference for EEEvis over PubMed as a biomedical literature search tool (91.7%). We believe this shows that EEEvis fulfills the design requirements as a literature search tool and improves the user experience.

EEEvis follows a progressive visual analytic approach to reduce the time delay during a large-scale query search and provide interactivity to the system. However, despite the users being informed that the search results of EEEvis are updated progressively and users can explore the results during the fetch process, most users waited until the fetch process was completed. There was no explicit feedback on why users hesitated to explore the results during the fetch process. We suppose that the search query results during the user study tasks were not big enough (684 ~ 2205 articles) to invoke a severe fetching delay, and users did not have the necessity of exploring the approximate partial batch results.

### Design implications

According to our findings, we present two implications for improving the design of visual analytics systems for biomedical literature search.

#### Provide overview for bibliographic features

While searching for optimal articles using EEEvis, every participant used the citation count and impact factor as key indicators for their search strategy. Novices may find it difficult to establish a search strategy when the search space is too wide and noisy. Conventional importance metrics, such as the citation counts and impact factor, may provide a cornerstone to explore the literature search space. Most of the participants identified the appropriate range of the values in which they were not interested with the bibliographic feature overviews and reduced the information noise of the search space by filtering out articles in which they were not interested. A total of 21 out of 24 participants pointed out that “Sorting and filtering by citation count and impact factor” was the main advantageous feature of EEEvis compared to PubMed.

#### Provide multiple features together

As novices often experience interpretation problems [24], presenting the search dataset with a single bibliographic feature at a time is not sufficient to provide a clear understanding. Presenting multiple bibliographic features in multiple coordinated views helped the users to understand the dataset more precisely. Users utilized the brushing and linking techniques to find specific regions of interest, and to reveal the connections among different bibliographic representations. Our findings suggest that biomedical literature search tools should provide different viewpoints of the data with multiple coordinated views.

## Limitations and future work

EEEvis is still a work in progress, which means that several features require improvement. These include improving the range selection accuracy and granularity of the range filters and enabling to save the user bookmarked(stared) articles. Furthermore, the information on the impact factors and citation counts should be updated from an external database periodically, as these are not provided by the PubMed API.

Artificial intelligence-based models can enhance the quality of the user experience in literature search systems. Conventional search systems like PubMed or EEEvis provide search results with ranking algorithms that rank the relevance of the documents from the search keyword [1]. However, content-based ranking results are usually not self-explanatory, and users will still need to seek through the text and bibliographic information to determine whether the documents are an appropriate match for them. With the attention mechanism-based language models and progress in the explainable AI field [25–28], it is possible to visualize which part of the text is responsible for the relevance rankings. We believe that making search results more explainable will provide intuitive visual cues, and users will be able to develop more efficient search strategies.

The study exhibits several limitations. First, the pre-development survey was conducted using a relatively small number of medical researchers with a limited background. Various additional opinions on PubMed or other search tools could be obtained with greater numbers and more diverse backgrounds, such as countries, mother languages, and ethnicities, of medical researchers.

Second, the results of the user study should be interpreted with caution. The negative results presented in this study do not suggest there be no true differences between the two systems. The number of subjects was small, the participants had various fields of expertise and areas of interest, and the queries that were presented to the subjects were individualized. This resulted in heterogeneity, which may have masked the true difference in performance between the two systems. Furthermore, the methods that were used to measure the performance of the systems represented only a part of the overall performance, as the performance of search systems can be measured by many other means [29,30]. However, this comparison can be meaningful to descriptively show that EEEvis and PubMed are not grossly different in their efficiency. In particular, these results indirectly show that EEEvis could be a user-friendly interface, as participants received orientation for only 10 minutes, whereas PubMed is an interface exposed to an average of 4 years.

Third, the task size might have been too small to observe different user behaviors between EEEvis and PubMed. In the case of PubMed, the median number of query results was 1,446, and the median task completion time was 306 seconds. As we supposed that the task size might be why users did not explore the dataset between progressive updates, different task sizes might affect user behaviors. So, conducting case studies with real-world scenarios that usually have more extensive search results and longer task time might reveal significant insights between systems.

Finally, comparing behaviors among populations with different biomedical literature search experiences might be an interesting approach. In the user study, we recruited participants with a similar amount of experience in biomedical literature searching to control the effect of the expert level. However, several participants commented that EEEvis would be more effective for novice users than expert users who have already established an optimal search strategy.

## Conclusions

In this study, we propose EEEvis, which is a novel interactive visual analytic system for a biomedical literature search task and a design guideline for the task. We demonstrated that the proposed system could improve the user experience in searching the appropriate literature by conducting a controlled user study. With the application of more future work, we expect EEEvis to become a system that can efficiently aid medical researchers in searching important articles of interest in the era of information overload.

## Supporting information

S1 Fig Filtering and focusing. (A) Filtering: the documents in the range of interest are filtered by dragging the mouse horizontally on each metadata histogram. Each histogram is coordinated and EEEVis supports a cross-filter function, so that the filter result (blue histogram) will be an intersection of each filter. (B) Focusing: focusing is a bushing and linking interaction. The interaction is similar to filtering; however, it is an independent filter function that does not alter the document subset of the filtering. This mouse interaction highlights the documents between the two coordinated views, the Timeline View, and the Author Network View.
(TIF)

S1 Table Preliminary Google survey. (DOCX)

S2 Table Two optimized queries for each participant. * All the participants were randomly allocated into two groups: E-P means the sequence of EEEvis to Pubmed, and P-E means the sequence of PubMed to EEEvis. † Query 1 assumes a situation where the participant makes a presentation on the ‘topic (A)’ at a specific conference. If you are unfamiliar with the topic (A), what key article would you find in that field to prepare for a presentation? For the same query, perform a literature search in the order of E-P or P-E. The time limit for each search engine is 10 minutes. § Query 2 assumes a situation where the participant writes an introduction or discussion part of a certain paper. If you were to select and use either ‘sentence (B)’ or ‘sentence (C)’ in a particular paragraph, which article would you cite (except participants 14)? For the same query, perform a literature search in the order of E-P or P-E. The time limit for each search engine is 10 minutes.
(DOCX)

S3 Table Step A data: Preliminary survey. (XLSX)

S4 Table Step C data: Randomized study results. (XLSX)
