# Supplementary figures and images for: Efficacy improvement in searching MEDLINE database using a novel PubMed visual analytic system: EEEvis
Source: PLoS One. 2023 Feb 9;18(2):e0281422. doi: 10.1371/journal.pone.0281422 (PMC9910730; doi:10.1371/journal.pone.0281422)

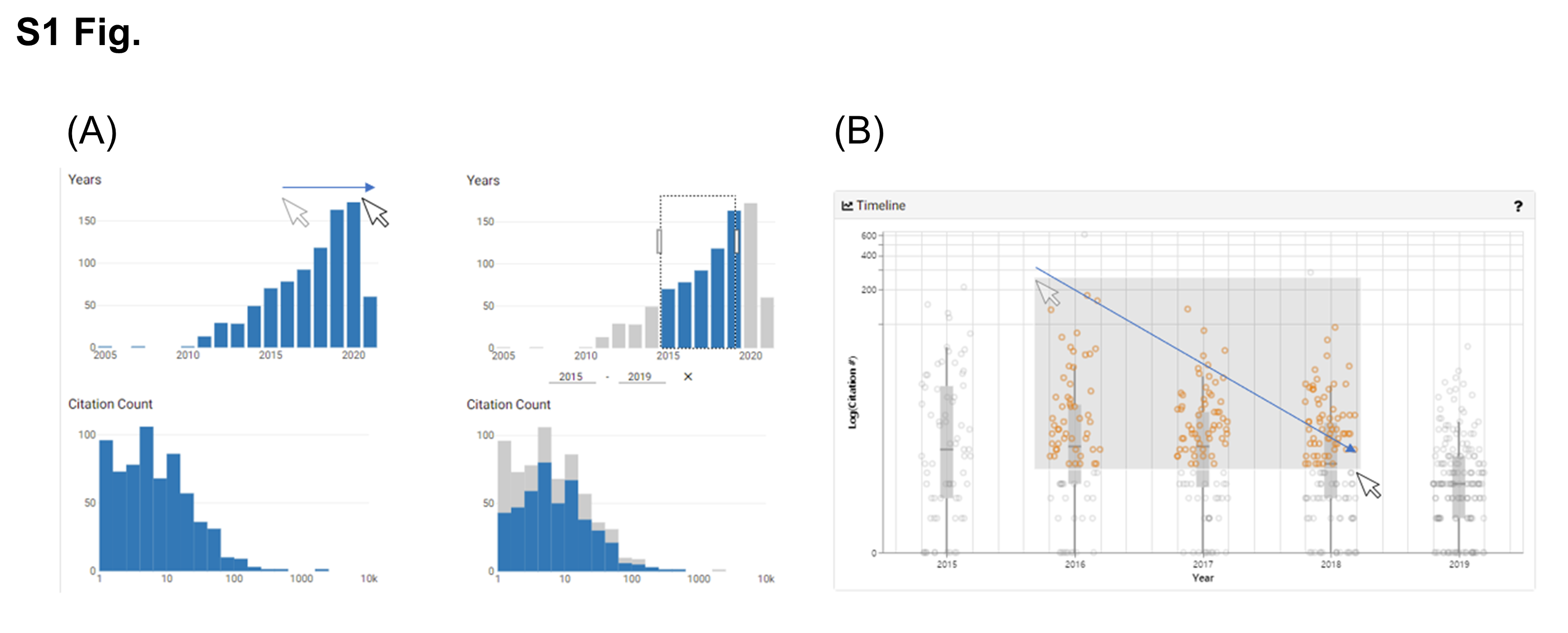

Supplement: S1 Fig — (A) Filtering: the documents in the range of interest are filtered by dragging the mouse horizontally on each metadata histogram. Each histogram is coordinated and EEEVis supports a cross-filter function, so that the filter result (blue histogram) will be an intersection of each filter. (B) Focusing: focusing is a bushing and linking interaction. The interaction is similar to filtering; however, it is an independent filter function that does not alter the document subset of the filtering. This mouse interaction highlights the documents between the two coordinated views, the Timeline View, and the Author Network View. (TIF) [file pone.0281422.s001.tif]
