# Supplementary material for: Efficacy improvement in searching MEDLINE database using a novel PubMed visual analytic system: EEEvis
Source: PLoS One. 2023 Feb 9;18(2):e0281422. doi: 10.1371/journal.pone.0281422 (PMC9910730; doi:10.1371/journal.pone.0281422)
Supplement: S1 Table — (DOCX) [file pone.0281422.s002.docx]

**S1 Table. Preliminary Google survey**

| **Section 1. Research experience** |
| --- |
| Q1. What is your area of expertise? (e.g. gastroenterology, neurology, radiology, pathology, preventive medicine, medical statistics, etc.) _______________________  Q2. What is your age?  1) Less than 30  2) 30 ~ 34  3) 35 ~ 39  4) 40 ~ 44  5) 45 ~ 50  6) 50 ~ 54  7) 55 ~ 59  8) 60 or more    Q3. What is your year of graduation from medical school? (e.g. 2008) ____________    Q4. How many research articles have you been significantly involved as the first author, the corresponding author, or co-author having invested 3 workdays or more? The papers may include unpublished research.  1) 0  2) 1  3) 2  4) 3~5  5) 6~10  6) 11~20  7) 21 or more |
| **Section 2. Literature search engine use experience** |
| Q1. On what purpose do you mainly use PubMed? (Multiple choices allowed)  1) Writing Article  2) Searching materials for seminar  3) Searching for knowledges (e.g. research updates, drug information)  4) Routine updates of literatures on subjects of interest (including My NCBI subscription)  5) Meta-analysis  6) Others ( )    Q2. What search engines do you use for medical literature search? (Multiple choices allowed)  1) PubMed  2) Embase  3) Google or Google Scholar  4) Scopus  5) Web of Science  6) Dbpia (in Korean database)  6) Others ( )    Q3. When using PubMed, how frequently do you use Boolean operators? (e.g. AND, OR, TI, TA, AU, AD, DP)  1) Almost always  2) Often  3) Sometimes  4) Rarely or never  5) I rarely use PubMed, so this question is not applicable to me.    Q4. When using PubMed, which part of the window do you usually look? (Please refer to the images for the letter indications)  1) I mostly look at B part, and rarely at A and C parts.  2) I mainly look at B part, but I often use the filter function of A part.  3) I mainly look at B part, but I often use the MeSH term or related article links of C part.  4) I mainly look at A or C parts, and rarely look at B part.  5) I mainly look at B parts, and look at other parts as needed.  6) I rarely use PubMed, so this question is not applicable to me.    Q5. When using PubMed, how do you use “Sort by” function located below the search bar?  1) I rarely use it.  2) I use it depending on the search results. (e.g. 50 abstracts per page)  3) I have the preset settings which I prefer and use frequently.  4) I rarely use PubMed, so this question is not applicable to me.    Q6. When using PubMed, which features of part B do you usually look at first? (Please refer to the images for the letter indications)  1) I skim at the article titles to look for the suitable article for the purpose of my search.  2) I look at the author names and look for the leading expertise on the subject of interest.  3) I look at the journal names and look for that with high impact factor.  4) I sort the results by “Best Match” and skim the results.  5) I let abstracts appear and search for the keywords by using control + F.  6) I rarely use PubMed, so this question is not applicable to me.    Q7. When using PubMed, do you use “Related Article” function?  1) I don’t know about the function.  2) I ignore the function and instead look for the results by using current keywords.  3) I use the function after I found the appropriate article.  4) I use it simultaneously with the current keywords.  5) I use it case by case.  6) I rarely use PubMed, so this question is not applicable to me.    Q8. How do you manage literature PDF files?  1) I download the reference PDF files when necessary and save them in computer folders.  2) I download the reference PDF files when necessary and save them in EndNote.  3) I download the reference PDF files when necessary but do not save them.  4) I routinely download the reference PDF files and save them in computer folders.  5) I routinely download the reference PDF files and save them in EndNote.  6) I do not download PDF files but organize the PMID or keywords in excel or word files.  7) There is no definite management pattern for me.  8) Others ( ) |
| **Section 3. Functions for new search engine** |
| Q1. Do you feel any shortcomings when using PubMed? You may write “None” if there aren’t any, and you may write multiple opinions.  ( )    Q2. What functions do you think need to be included in a new search engine? (Upto 3 choices possible)  1) Visualization of interaction between articles by keywords  2) Sorting the articles by journal impact factor  3) Sorting the articles by citation counts  4) Searching by the leading expertise of the subject  5) Visualization of articles on the subject by timeline  6) Automatic word completion function (e.g. AND, OR)    Q3. Is there any other function would you want a new search engine to include that is not included in the above question? You may write “None” if there aren’t any, and you may write multiple opinions.  ( ) |
