# Supplementary material for: Efficacy improvement in searching MEDLINE database using a novel PubMed visual analytic system: EEEvis
Source: PLoS One. 2023 Feb 9;18(2):e0281422. doi: 10.1371/journal.pone.0281422 (PMC9910730; doi:10.1371/journal.pone.0281422)
Supplement: S2 Table — * All the participants were randomly allocated into two groups: E-P means the sequence of EEEvis to Pubmed, and P-E means the sequence of PubMed to EEEvis. † Query 1 assumes a situation where the participant makes a presentation on the ‘topic (A)’ at a specific conference. If you are unfamiliar with the topic (A), what key article would you find in that field to prepare for a presentation? For the same query, perform a literature search in the order of E-P or P-E. The time limit for each search engine is 10 minutes. § Query 2 assumes a situation where the participant writes an introduction or discussion part of a certain paper. If you were to select and use either ‘sentence (B)’ or ‘sentence (C)’ in a particular paragraph, which article would you cite (except participants 14)? For the same query, perform a literature search in the order of E-P or P-E. The time limit for each search engine is 10 minutes. (DOCX) [file pone.0281422.s003.docx]

**S2 Table. Two optimized queries for each participant**

| **No** | **Subspecialty** | **Group**^*^ | **Query 1**^†^ | **Query 2** ^§^ |
| --- | --- | --- | --- | --- |
| 1 | Gastroenterology | P-E | (A) Are there any benefits using ursodeoxycholic acid after cholecystectomy? | (B) *Helicobacter pylori* infection is a definite risk factor for gastric cancer. VS. (C) It is controversial whether *Helicobacter pylori* infection is a risk factor for gastric cancer. |
| 2 | Gastroenterology | P-E | (A) Which regimen would be the best second-line chemotherapy for patients who progressed on lenvatinib? | (B) *Helicobacter pylori* infection is a definite risk factor for gastric cancer. VS. (C) It is controversial whether *Helicobacter pylori* infection is a risk factor for gastric cancer. |
| 3 | Gastroenterology | P-E | (A) What are the systemic treatment regimens available for patients with pancreatic neuroendocrine tumor? | (B) Neoadjuvant chemotherapy is beneficial for patients with pancreatic cancer. VS (C) It is controversial whether neoadjuvant chemotherapy is beneficial for patients with pancreatic cancer. |
| 4 | Hematology-Oncology | P-E | (A) What are the systemic treatment regimens available for patients with pancreatic neuroendocrine tumor? | (B) Neoadjuvant chemotherapy is beneficial for patients with pancreatic cancer. VS (C) It is controversial whether neoadjuvant chemotherapy is beneficial for patients with pancreatic cancer. |
| 5 | Gastroenterology | E-P | (A) What are the systemic treatment regimens available for patients with pancreatic neuroendocrine tumor? | (B) Neoadjuvant chemotherapy is beneficial for patients with pancreatic cancer. VS (C) It is controversial whether neoadjuvant chemotherapy is beneficial for patients with pancreatic cancer. |
| 6 | Gastroenterology | P-E | (A) Which regimen would be the best second-line chemotherapy for patients who progressed on lenvatinib? | (B) *Helicobacter pylori* infection is a definite risk factor for gastric cancer. VS. (C) It is controversial whether *Helicobacter pylori* infection is a risk factor for gastric cancer. |
| 7 | Gastroenterology | E-P | (A) What are the systemic treatment regimens available for patients with pancreatic neuroendocrine tumor? | (B) Neoadjuvant chemotherapy is beneficial for patients with pancreatic cancer. VS (C) It is controversial whether neoadjuvant chemotherapy is beneficial for patients with pancreatic cancer. |
| 8 | Gastroenterology | P-E | (A) What are the possible systemic treatment options for the patients with biliary tract cancer who progressed on the combination of gemcitabine and cisplatin? | (B) Neoadjuvant chemotherapy is beneficial for patients with pancreatic cancer. VS (C) It is controversial whether neoadjuvant chemotherapy is beneficial for patients with pancreatic cancer. |
| 9 | Gastroenterology | P-E | (A) What is the relationship between ulcerative colitis and colorectal cancer? | (B) *Helicobacter pylori* eradication for infected patients prevents stomach cancer development. VS. (C) It is controversial whether *Helicobacter pylori* eradication for infected patients prevents stomach cancer development. |
| 10 | Cardiology | E-P | (A) What is the appropriate duration of dual antiplatelet therapy for patients who underwent percutaneous coronary intervention? | (B) It is recommended for clinicians to start angiotensin receptor-neprilysin inhibitor for patients with acute decompensated reduced ejection fraction heart failure at the time of admission. VS. (C) It is controversial whether angiotensin receptor-neprilysin inhibitor should be started for patients with acute decompensated reduced ejection fraction heart failure at the time of admission. |
| 11 | Gastroenterology | E-P | (A) How many days are required for patients to stop antiplatelet or anticoagulation agents before undergoing endoscopic submucosal dissection for early gastric cancer? | (B) Intestinal metaplasia is a risk factor for gastric cancer. VS (C) It is controversial whether intestinal metaplasia is a risk factor for gastric cancer. |
| 12 | Gastroenterology | E-P | (A) Which regimen would be the best second-line chemotherapy for patients who progressed on lenvatinib? | (B) Intestinal metaplasia is a risk factor for gastric cancer. VS. (C) It is controversial whether intestinal metaplasia is a risk factor for gastric cancer. |
| 13 | Nephrology | P-E | (A) Which one of parenteral or oral iron replacement is better for patients with chronic kidney disease? | (B) There are survival benefits of early formation of arteriovenous fistula in patients with end-stage renal disease who initiated hemodialysis. VS. (C) It is controversial whether there are survival benefits of early formation of arteriovenous fistula in patients with end-stage renal disease who initiated hemodialysis. |
| 14 | Obstetrics-Gynecology | P-E | (A) What is the optimal antibiotic regimen for intrauterine infection? | You are working on an article and planning to write a sentence on the first line optimal chemotherapeutic agent for mucinous type ovarian cancer. |
| 15 | Hematology-Oncology | E-P | (A) What is the optimal first-line treatment for chronic myeloid leukemia? | (B) Diagnosis of smoldering multiple myeloma is an indication for treatment initiation. VS (C) It is controversial whether the diagnosis of smoldering multiple myeloma is an indication for treatment initiation. |
| 16 | Hematology-Oncology | E-P | (A) What is CAR-T cell therapy and what are the indications? | (B) Tumor mutation burden is a biomarker for immune checkpoint inhibitor response. VS. (C) It is controversial whether tumor mutation burden is a biomarker for immune checkpoint inhibitor response. |
| 17 | Pulmonology | P-E | (A) What is the indication of remdesivir for COVID-19? | (B) Steroid administration improves survival in patients with acute exacerbation of idiopathic pulmonary fibrosis. VS. (C) It is controversial whether steroid administration improves survival in patients with acute exacerbation of idiopathic pulmonary fibrosis. |
| 18 | Hematology-Oncology | E-P | (A) What are the treatment options for thrombotic thrombocytopenic purpura? | (B) In patients with hormone receptor positive breast cancer, the recommended duration of adjuvant hormone therapy is 5 years. VS (C) It is controversial on the duration of adjuvant hormone therapy for patients with hormone receptor positive breast cancer. |
| 19 | Thoracic surgery | E-P | (A) What are the long-term complications in patients with left ventricular assist devices? | (B) Artery graft shows superior outcome compared with venous graft in coronary artery bypass graft surgery. VS. (C) It is controversial whether artery graft shows superior outcome compared with venous graft in coronary artery bypass graft surgery. |
| 20 | Pediatrics | E-P | (A) What is the biomarker used for monitoring acute kidney injury in premature infants? | (B) Younger children are more susceptible to urinary tract infection by ESBL-producing bacteria. VS. (C) It is controversial whether younger children are more susceptible to urinary tract infection by ESBL-producing bacteria. |
| 21 | Orthopedic surgery | P-E | (A) What are the novel treatment options for patients with osteosarcoma? | (B) Pelvic angioplasty showed superior outcome compared with peritoneal pelvic packing in pelvic fracture patients with hemodynamic instability. VS. (C) It is controversial whether pelvic angioplasty is superior outcome compared with peritoneal pelvic packing in pelvic fracture patients with hemodynamic instability. |
| 22 | Anesthesiology | E-P | (A) Which one of inhalation or intravenous anesthesia is better for one-lung ventilation? | (B) Propofol has more protective effect on postoperative acute kidney injury compared with inhalant agents. VS. (C) It is controversial whether propofol has more protective effect on postoperative acute kidney injury compared with inhalant agents. |
| 23 | Hepatobiliary surgery | P-E | (A) What are the effects of preoperative biliary tract drainage on prognosis according to the location of extrahepatic biliary cancer? | (B) Pancreatic enzyme medication reduces the risk of fatty liver in patients who had pancreatectomy. VS. (C) It is controversial whether pancreatic enzyme medication reduces the risk of fatty liver in patients who had pancreatectomy. |
| 24 | Gastric surgery | P-E | (A) What are the surgical options for patients with stomach cancer and single liver metastasis? | (B) Long term fat-soluble vitamin supplements are necessary for patients who underwent bariatric surgery. VS. (C) It is controversial whether long term fat-soluble vitamin supplements are necessary for patients who underwent bariatric surgery. |

* All the participants were randomly allocated into two groups: E-P means the sequence of EEEvis to Pubmed, and P-E means the sequence of PubMed to EEEvis.

† Query 1 assumes a situation where the participant makes a presentation on the ‘topic (A)’ at a specific conference. If you are unfamiliar with the topic (A), what key article would you find in that field to prepare for a presentation? For the same query, perform a literature search in the order of E-P or P-E. The time limit for each search engine is 10 minutes.

§ Query 2 assumes a situation where the participant writes an introduction or discussion part of a certain paper. If you were to select and use either ‘sentence (B)’ or ‘sentence (C)’ in a particular paragraph, which article would you cite (except participants 14)? For the same query, perform a literature search in the order of E-P or P-E. The time limit for each search engine is 10 minutes.
